# A Novel Water-Soluble Polysaccharide from Daylily (*Hemerocallis citrina* Baroni): Isolation, Structure Analysis, and Probiotics Adhesion Promotion Effect

**DOI:** 10.3390/foods13050721

**Published:** 2024-02-27

**Authors:** Qinfei Ke, Hui Wang, Yuan Xiao, Xingran Kou, Feng Chen, Qingran Meng, Wenjie Gao

**Affiliations:** 1 Collaborative Innovation Center of Fragrance Flavour and Cosmetics, School of Perfume and Aroma Technology, Shanghai Institute of Technology, Shanghai 201418, China; kqf@sit.edu.cn (Q.K.); wh1999laura@163.com (H.W.); kxran@163.com (X.K.); 2 School of Public Health, Wannan Medical College, Wuhu 241002, China; xiaoyuan@wnmc.edu.cn; 3 Department of Food, Nutrition, and Packaging Sciences, Clemson University, Clemson, SC 29634, USA; fchen@clemson.edu; 4 School of Ecological Technology and Engineering, Shanghai Institute of Technology, Shanghai 201418, China

**Keywords:** *Hemerocallis citrina* Baroni, water-soluble polysaccharide, structure analysis, PTIO-scavenging activity, probiotic adhesion

## Abstract

The daylily (*Hemerocallis citrina* Baroni) flower is a traditional raw food material that is rich in a variety of nutrients. In particular, the content of polysaccharides in daylily is abundant and has been widely used as a functional component in food, cosmetics, medicine, and other industries. However, studies on the structure-effective relationship of daylily flower polysaccharides are still lacking. In view of this, daylily flower polysaccharides were isolated and purified, and their physical and chemical properties, structure, antioxidant activity, and adhesion-promoting effect on probiotics were evaluated. The results showed that a novel water-soluble polysaccharide (DPW) with an average molecular weight (*M*_w_) of 2.224 kDa could be successfully isolated using column chromatography. Monosaccharide composition analysis showed that DPW only comprised glucose and fructose, with a molar ratio of 0.242:0.758. Through methylation and nuclear magnetic resonance (NMR) analysis, it was inferred that DPW belonged to the fructans group with a structure of α-D-Glcp-1→2-β-D-Fruf-1→(2-β-D-Fruf-1)_n_→. Antioxidant analysis showed that DPW showed strong 2-Phenyl-4,4,5,5-tetramethylimidazoline-1-oxyl-3-Oxide (PTIO-scavenging activity with IC_50_ of 1.54 mg/mL. DPW of 1.25 to 5 mg/mL could significantly increase the adhesion rate of *Lactobacillus acidophilu*, *Lactobacillus casei*, *Bifidobacterium adolescentis*, and *Lactobacillus plantarum* on Caco-2 cells. Considering the above results, the present study provides a theoretical basis and practical support for the development and application of daylily polysaccharides as a functional active ingredient.

## 1. Introduction

*Hemerocallis citrina* Baroni, also known as daylily, is a tall angiosperm of the *Hemerocallis* genus in the *Asphodelaceae* Juss family. It is a treasure of flower horticulture with very high ornamental value worldwide, especially in decorating gardens or flower borders [1]. Furthermore, daylily flowers are rich in bioactive substances including carotenoids, vitamins, flavonoids, catechins, and polysaccharides (soluble and insoluble dietary fiber) [2], and they have been widely planted and eaten as a nutritious food product in East and Southeast Asia, especially in China, India, Korea, Japan, Thailand, and so on [3]. Since daylily flowers age quickly and can be predicted (after blooming, the daylily flower will wither within a day), they are usually steamed, dried, and processed into dried vegetables. In addition, daylily also has high medicinal value, with properties such as reducing inflammation [4], inhibiting cancer cell proliferation [5], improving sleep [6,7], resisting oxidation [2,8], and relieving depression [9], and has been used as a traditional medicine worldwide. In particular, daylily flower water extracts, mainly polysaccharides, have been included in the International Cosmetic Ingredient Dictionary and Handbook as a functional cosmetic ingredient.

Polysaccharides, a typical biomacromolecule, are widely distributed in higher plants, algae, fungi, and animals [10], and are the most abundant biopolymers in nature, with many functions including energy storage, structural support, and defense. Structurally, polysaccharides consist of more than 10 identical or different monosaccharides polymerized by α- or β-glycosidic bonds. Many researchers have demonstrated that plant polysaccharides have various biological activities, such as immunomodulatory activity, antidiabetic activity, anti-metastatic activity, hypoglycemic activity, antioxidant activity, and apoptosis-inducing activity against cancer cells [11,12,13]. In addition to being used as functional active ingredients in foods, plant polysaccharides are widely used in medicine, light industry, agriculture [14], and other fields.

Probiotics, which can improve the host’s intestinal microecological balance and contain physiologically viable bacteria, have been widely used in food, health care products, drugs, and clinical treatment [15]. In order to play a probiotic role, probiotics must maintain a certain number in the gastrointestinal tract, and they adhere to the digestive tract mucosa of the human body through some specific components on the surface (colonization), rejecting the adhesion and invasion of normal cells by pathogenic bacteria [16]. Adhesion to the mucosa is the first step in colonization and proliferation. The adhesion ability of probiotics can provide them with a competitive advantage and is a necessary condition for their long-term colonization in the digestive tract [17]. Therefore, the research and development of different sources of polysaccharides or oligosaccharides with prebiotic effects is of great significance for reducing the production cost of prebiotics and meeting different health needs [18].

For polysaccharides from different sources, the chemical structure determines their biological activities, wherein the molecular shape, molecular main chains, relative molecular mass, degree of branching, molecular chemical modifications, and so on significantly affect their biological activities [19,20]. Therefore, it is crucial to explore the structure-effective relationship of specific polysaccharides, which can help the primary screening of the efficacy of having the same structural components. For instance, most glucans with tumor inhibitory activity are dominated by (1→3)-β-D glucans, and the glucose residues are distributed along the main chain randomly. Such comb structure exerts antitumor activity by the host immune system [21]. In addition, Jing et al. (2023) found that galactose in the monosaccharide composition as well as the molecular weight showed a positive correlation effect on free radical-scavenging activity of different-sourced *Salvia miltiorrhiza* polysaccharides [22].

In our previous research, ultrasonic-assisted extraction was successfully used to extract daylily polysaccharides (DP) with an extraction yield of 15.25% under optimal extraction conditions. Through DEAE-Sepharose column chromatography, four new water-soluble fractions, eluted with double-distilled water and sodium chloride solution (0.2, 0.5, and 1.0 mol/L), were successfully obtained with obvious 1,1-Diphenyl-2-picrylhydrazyl radical 2,2-Diphenyl-1-(2,4,6-trinitrophenyl)hydrazyl (DPPH), 2,2′-azino-bis(3-ethylbenzothiazoline-6-sulfonic acid) (ABTS), hydroxyl free radical-scavenging activity, and ferric-reducing antioxidant power [23]. The above study only evaluated the efficacy of crude DP, but from the perspective of the structure-effective relationship, it is necessary to analyze the structure in depth, which would be of great benefit to the high-value utilization of the effective components of DP.

In the present study, structure identification of the water-elution fraction (DPW) was first studied using column chromatography coupled with Fourier-transform infrared spectroscopy (FT-IR) analysis, and one-dimensional and two-dimensional nuclear magnetic resonance (1D NMR and 2D NMR) analysis. Then, the antioxidant activity and the adhesion effect of DPW treatment on probiotics on the cell surface were evaluated. The results of this study aim to provide a theoretical basis and practical support for the development and application of daylily polysaccharides as a functional active ingredient.

## 2. Materials and Methods

### 2.1. Materials

Dried daylily buds with a moisture content of ~15% were produced in Qidong County, Hunan Province 421600, China. The obtained materials were dried in an air blast-drying oven at 60 °C to a moisture content of ~8%. Then, the dried materials were crushed (800 A multi-function pulverizer, Yongkang Jinsui Machinery Factory, Jinhua, China) and sieved using an 80-mesh standard sieve (Haoquan screen factory, Shaoxing, China), and the fine daylily powder samples were packaged in Ziplock bags and stored in desiccators for further analysis.

### 2.2. Chemicals and Microorganisms

PTIO· (CAS: 18390-00-6, >98.0%), fetal bovine serum, and minimum essential medium (MEM) culture medium were purchased from Sigma Alrich (Shanghai, China). Monosaccharides and glucuronic acid standards, Sephadex G-30, methylation kit, and a DEAE-Sepharose Fast Flow column (2.6 cm × 60 cm) were provided by Borui Saccharide Biotech Co., Ltd. (Yangzhou, China). Sodium borohydride and dextrans (5000, 11,600, 23,800, 48,600, 80,900, 148,000, 273,000, 409,800, and 670,000 Da) with a purity of ≥97% were purchased from Merck Limited (Shanghai, China). *Lactobacillus acidophilus* (ATCC 4356), *Lactobacillus casei* (ATCC 393), E305 *Bifidobacterium adolescentis* (ATCC 15704), *Lactobacillus plantarum* (ATCC 8014), and Caco-2 were obtained from Beijing Biobw Biotechnology Co., Ltd. (Beijing, China). MRS culture medium, D_2_O, deuterated acetone, dialysis bag (MWCO 3500 Da), and other reagents and chemicals of analytical grade were purchased from Titan Technology Co., Ltd. (Shanghai, China).

### 2.3. DP Extraction and Purification

The extraction procedure was carried out according to our previous protocol [23], with minor modifications. In brief, the daylily powder sample was first pretreated with petroleum ether (1:10 (*w*/*v*, g/mL) three times, followed three times by 80% ethanol aqueous solution (1:10 (*w*/*v*, g/mL) to remove impurities. The mixture was then filtered, dried, and extracted with deionized water under optimal conditions [23]. The extracts were centrifuged, concentrated, decolorized, and alcohol-precipitated to obtain crude DP. Then, the precipitate was dissolved in deionized water and treated with a mixture of chloroform and n-butanol (4:1, *v*:*v*) several times to remove proteins. The remaining aqueous fraction was collected and dialyzed (typical molecular weight cut-off = 500 Da) for 72 h (at 4 °C), during which time the deionized water (DI water) was changed every 4 h. The dialysate product was reprecipitated with absolute ethanol, centrifugated, and lyophilized (at −54 °C) to obtain the preliminarily purified DP.

The above purified DP were dissolved in water again and further fractionated with a Borui DEAE-Sepharose Fast Flow column. After loading, the chromatographic system was eluted with double-distilled water and NaCl solution (0.2, 0.5, and 1.0 mol/L) successively. During elution, the eluate was collected every 4 mL, and the polysaccharide content in each tube was detected using the phenol-sulfuric acid method [24]. Meanwhile, the elution curve was drawn based on the polysaccharide content in each tube, and the eluted fractions were collected and combined based on the peaks in the elution curve. Each combined fraction was dialyzed to remove NaCl, precipitated with anhydrous ethanol, and freeze-dried, and finally four polysaccharide fractions were obtained: the water elution fraction (DPW) and three NaCl elution fractions (Figure S1). In the present study, mainly the DPW with the highest content was studied further.

The DPW fraction was dissolved in DI water. After thorough mixing, the mixture was centrifuged at 12,000 rpm for 10 min (H2050R, Xiangyi Centrifuge Instrument Co., Ltd., Changsha, China). The obtained supernatant was further analyzed using a Borui Saccharide auto-purify gel permeation chromatography system combined with an online RI-502 SHODEX refractive index detector (RID, Yangzhou, China). The symmetrical peaks were collected and condensed using a rotary evaporator. The concentrate was then freeze-dried to obtain a purified DPW fraction.

### 2.4. Molecular Weight Determination

The purified DPW and standard dextrans were accurately weighed and dissolved separately in DI water to prepare stock solutions (5 mg/mL). After centrifuging (12,000 rpm, 10 min), the supernatant was collected and filtered with 0.22 μm nylon filters. The molecular weight of DPW was tested using an HPLC-RID (LC-10A, Shimadzu (Shanghai) Global Laboratory Consumables Co., Ltd., Shanghai, China) coupled with a BRT105-104-102 tandem gel column (8 mm × 300 mm) (Borui Saccharide Biotech Co., Ltd., Yangzhou, China). Regarding the chromatographic analysis conditions, 0.05 mol/L of NaCl solution was used as a mobile phase with a flow rate of 0.6 mL/min, and the column oven was maintained at 40 °C; the DPW injection volume was 20 μL.

### 2.5. Monosaccharide Analysis

The purified DPW was accurately weighed (5 mg) and hydrolyzed at 120 °C with trifluoroacetic acid (10 mL 2 mol/L) for 3 h, and the hydrolysis solution was collected and moved into a concentration vial and dried with an LC-DCY-12G Termovap sample concentrator (Lichen Technology, Shanghai, China). The remaining residue was redissolved in DI water (50 mL). The mixture was thoroughly mixed and centrifuged (12,000 rpm for 5 min) and the supernate was filtered with a 0.22 μm nylon filter before chromatographic analysis. A pulse amperometric detector and a Dionex CarbopacTMPA20 column (3 mm × 150 mm) coupled with an ICS5000 ion chromatography system (Thermo Fisher Scientific Inc., Shanghai, China) were used for monosaccharide composition. The chromatographic analysis conditions were as follows: 250 mmol/L of NaOH was used as mobile phase A, DI water was used as mobile phase B, and a mixture of 50 mmol/L of NaOH and 500 mmol/L CH_3_COONa was used as mobile phase C; the flow rate was kept at 0.3 mL/min, and the analysis temperature was maintained at 30 °C throughout the analysis.

### 2.6. Fourier-Transform Infrared Spectroscopy (FT-IR) Analysis

The FT-IR spectrum of the purified DPW was determined using an IRTracer-100 spectrophotometer (Shimadzu (Shanghai) Global Laboratory Consumables Co., Ltd.). In brief, 2 mg of purified DPW fraction was accurately weighed and ground with ~200 mg of dried KBr. The fine powder mixture was then pressed into a pellet and scanned (400–4000 cm^−1^). The potassium bromide slice without the DPW sample was used as a control.

### 2.7. Methylation Analysis

The methylation experiment with DPW was performed based on the method described previously [13]. An aliquot of 3 mg of DPW samples was dissolved in dimethyl sulfoxide (1 mL) in a glass reaction bottle. Then, solution A (anhydrous alkaline solution) in the methylation kit was quickly added. The mixture was sealed and dissolved under the action of ultrasound, and solution B (methyl iodide solution) in the methylation kit was added. After thorough mixing, the reaction system was incubated at 30 °C for 60 min under magnetic stirring. Finally, 2 mL of DI water was used to cessate the methylation reaction. Subsequently, methylated DPW was extracted with chloroform and hydrolyzed with trifluoroacetic acid (1 mL, 2 mol/L) for 90 min. The organic phase was then evaporated to dryness using a Termovap sample concentrator, and 2 mL of DI water and 60 mg of NaBH_4_ were added for a reduction reaction for 8 h. After neutralization with CH_3_COOH, the mixture was dried again and acylated with 1 mL of acetic anhydride (100 °C for 1 h). After the acylation reaction, the mixture was cooled in an ice bath, and an aliquot of 5 mL of methylbenzene was added and then evaporated to dryness to remove excess acetic anhydride. The above procedure was repeated at least five times. The obtained acetylated product was then dissolved in 2.5 mL of dichloromethane, and DI water of the same volume was added. After thorough mixing, the aqueous phase was discarded (repeated at least three times). The organic phase was collected and dried with a certain amount of anhydrous Na_2_SO_4_ and then fixed to 10 mL.

The resulting samples were analyzed with a QP2010 gas chromatography-mass spectrometer (GC-MS) (Shimadzu (Shanghai) Global Laboratory Consumables Co., Ltd.) equipped with an Rxi-5Sil MS capillary column (30 m × 0.25 mm × 0.25 μm). The temperature of the column oven was increased from 120 °C to 250 °C at a rate of 3 °C/min and then maintained for 5 min; helium (1 mL/min) was used as carrier gas. The temperature of the vaporizing chamber was 250 °C and the injection volume was 1 μL. The MS parameters were programmed as follows: scan modal with scan speed at 769; ionization energy was 300 eV; mass range was from 40 to 400 *m*/*z*. The detector started to detect after 5 min and ended at 40 min.

### 2.8. Nuclear Magnetic Resonance (NMR) Analysis

An aliquot of 30 mg of the purified DPW was first dissolved in D_2_O (0.5 mL) and freeze-dried (repeated two times). Then, the residue was redissolved in 0.5 mL of D_2_O again. After thoroughly dissolving, the obtained sample was transferred into an NMR tube and placed in a 600 MHz NMR instrument (Bruker Ascend 600, Bruker Corp, Fallanden, Switzerland) at 25 °C for 1D and 2D NMR analysis.

### 2.9. PTIO Free Radical-Scavenging Activity

PTIO·-scavenging activity of DPW was carried out based on the method described previously [25], with some modifications. One milliliter of each DPW solution (0, 0.125, 0.25, 0.5, 1.0, 2.0, and 4.0 mg/mL in DI water) and 1.0 mL of freshly prepared PTIO·(0.05 mg/mL, dissolved in 50 mM pH 7.4 phosphate buffer saline, PBS) were fully mixed and then incubated at 37 °C for 2 h. The absorbance was tested at 557 nm. PTIO·-scavenging activity was calculated accordingly, and Vitamin C (V_C_) was used as the control. Meanwhile, the half maximal inhibitory concentration (IC_50_) of DPW was calculated according to the inhibition percentage against the sample concentration.

### 2.10. Probiotic Strains Adhesion Analysis

DPW solutions (40, 20, 10, 5, 2.5, 1.25, 0.625, and 0.3125 mg/mL in DI water) were prepared and filtered using 0.22 μm microporous filter film to remove bacteria and then stored at 4 °C for further use. The four test strains of the logarithmic growth stage were diluted into 5 × 10^9^ CFU/mL with MEM medium. A total of 2 mL of Caco-2 cells (1 × 10^5^ cells/mL) in the logarithmic growth stage was moved into a sterile six-well plate and then cultured in a cell incubator (Thermo Fisher Scientific Inc., Shanghai, China) at 37 °C and 5% CO_2_ until the cell density reached ~80%. Then, the culture medium was removed, and the six-well plate was washed with fresh MEM medium twice. A total of 2 mL of the above prepared probiotic strains suspension was added into each well, and 1 mL of the DPW solution above was added. The cell, probiotic, and DPW were co-cultured in a cell incubator (37 °C and 5% CO_2_) for 2 h. After that, the culture medium was discarded, and the co-culture was cleaned with phosphate buffer saline (PBS) (pH 7.3) three times. The adherent bacteria on Caco-2 were fixed with 4% paraformaldehyde for 20 min, and then Gram staining was performed to count the adherent probiotic strains on cells under a microscope. The number of adherent probiotic strains on each Caco-2 cell (CFU/cell) was calculated.

### 2.11. Statistical Analysis

All the above experiments were conducted at least in triplicate, and the results were expressed as mean ± standard deviation (SD). The significance analysis was conducted based on the analysis of variance (ANOVA) and Duncan’s multiple range comparison test, with a probability level of *p* < 0.05 being considered statistically significant.

## 3. Results and Discussion

### 3.1. Isolation and Purification of DPs

In the present study, a total of four DPW fractions were eluted successfully (Figure S1): the water elution fraction (DPW) and 0.2, 0.5, and 2.0 mol/L NaCl elution fractions. The yields of each fraction were 34%, 11.1%, 6.7%, and 4%, respectively. Hence, DPW with the highest content was studied further.

**Figure 1:**
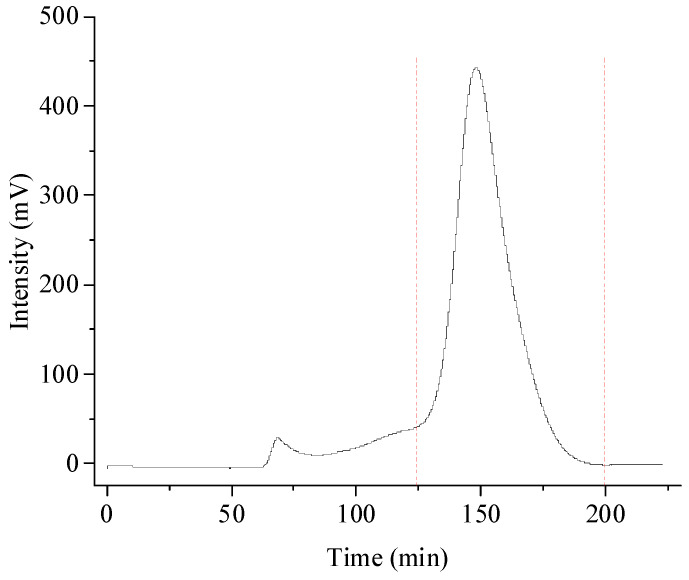
Purification of DPW Sephadex G-30 column.

For the Sephadex G-30 gel permeation chromatography, one main fraction was eluted (Figure 1). Eluates at 125–200 min as marked in red line were collected, concentrated, and lyophilized to obtain purified DPW for further analysis. The recovery rate, that is, the dry weight of DPW after purification/dry weight of total DPW before purification, was 65%.

### 3.2. Molecular Weight Analysis

**Figure 2:**
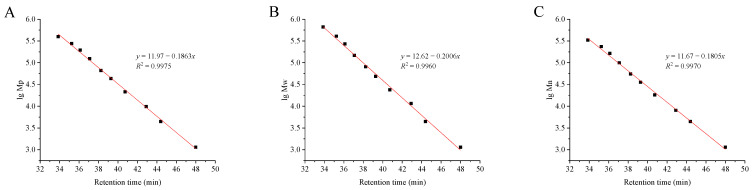
Calibration curves of standard dextrans. (**A**) for peak molecular weight; (**B**) for weight-average molecular weight; and (**C**) for number-average molecular weight.

**Figure 3:**
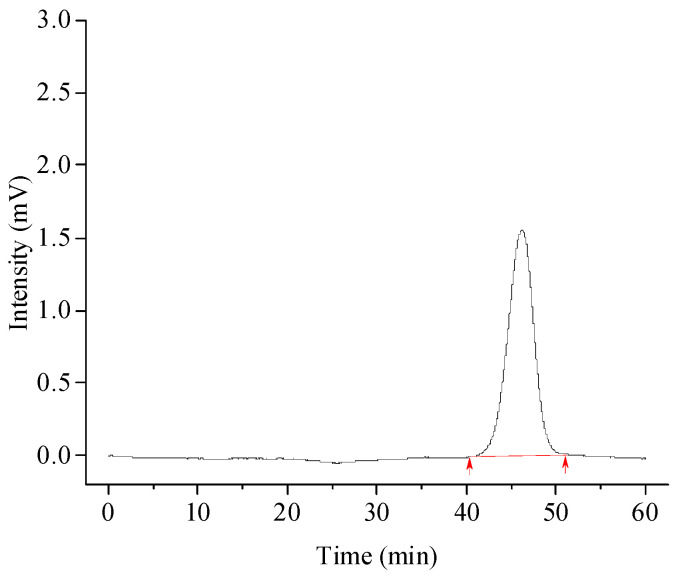
Molecular weight chromatogram of DPW.

Molecular weight is a very important parameter of polysaccharides and other biomacromolecules and is often closely related to many characteristics. Especially in biological research, the difference in the molecular weight of polysaccharides often leads to different biological and functional activities [26]. According to the high-performance size-exclusion chromatography (HPSEC) in the present study, the weight-average molecular weight (*M*_w_), the peak molecular weight (*M*_p_), and the number-average molecular weight (*M*_n_) of DPW were calculated based on three standard dextrans curves, that is, $\log_{10}M_{p}=-0.1836RT+11.967,\;R^{2}=0.9975$; $\log_{10}M_{w}=-0.2006RT+12.618,\;R^{2}=0.996$; and $\log_{10}M_{n}=-0.1805RT+11.673,\;R^{2}=0.997$ (Figure 2). As shown in Table 1 and Figure 3 (integration region was marked in red), the *M*_p_, *M*_w_, and *M*_n_ of DPW were calculated to be 2.275, 2.224, and 2.143 kDa, respectively.

The polydispersity index (PDI) or heterogeneity index, a characterization of the molecular weight distribution of a given polymer, is generally calculated as the ratio of *M*_w_ and *M*_n_. The smaller the PDI value, the more uniform the molecular weight of the polymer, and vice versa [24]. The DPW in the present study had a relatively low PDI, which was only 1.038 (Table 1).

### 3.3. Monosaccharide Composition Analysis

**Figure 4:**
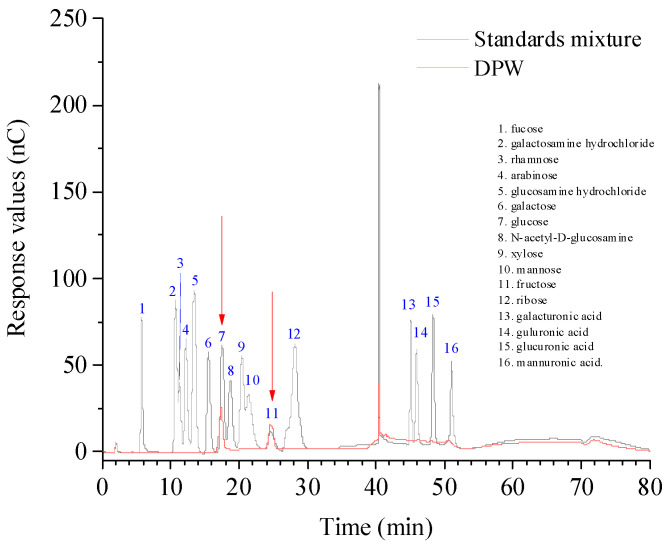
IC profiles of monosaccharide standards mixture and DPW.

As shown in Figure 4 and Table 1, the monosaccharide composition of DPW was relatively simple. It was only composed of two monosaccharides, that is, glucose and fructose as pointed out in red, and the molar ratio of the two monosaccharides was 0.242:0.758, based on the standard monosaccharides.

### 3.4. FT-IR Spectroscopy Analysis

**Figure 5:**
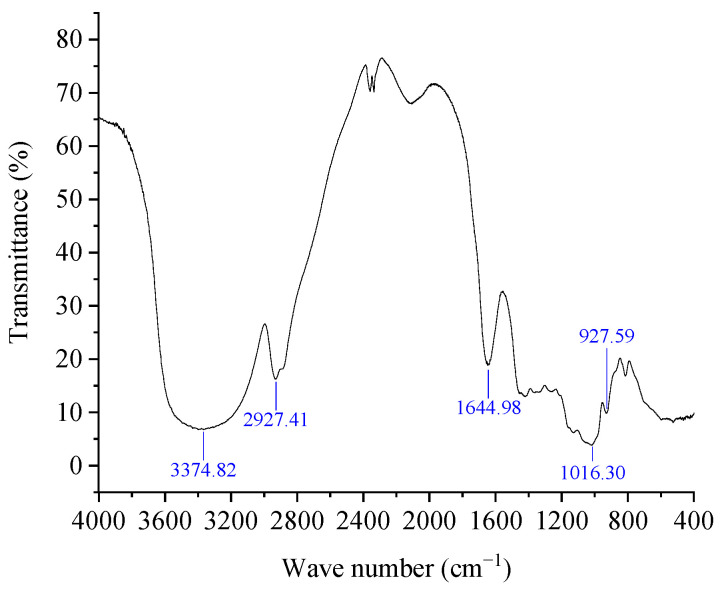
FTIR spectra of DPW.

The characteristic organic groups in polysaccharides can be preliminarily identified by FT-IR spectroscopy. As can be seen from Figure 5, the strong absorption band at 3374 cm^−1^ was categorized as -OH group stretching, and this typical absorption peak was commonly regarded as sugars or water [27]. According to Fuentes-Grünewald et al. (2015), the absorption peak at 2927 cm^−1^ was classified as the stretching vibration of C-H [28]. The absorption peak at 1644 cm^−1^ may be grouped into the absorption peak of crystal water [29]. The absorption peaks at 1016 cm^−1^ and 927 cm^−1^ were sorted into C-O stretching [30] and asymmetric ring stretching vibration [24], respectively.

### 3.5. Methylation Analysis

Polysaccharides contain a good deal of free hydroxyl groups. After the methylation reaction, these hydroxyl groups will generate methyl ether. When the glycosidic bond of the methylated polysaccharide is hydrolyzed, partially methylated monosaccharide components can be obtained. By analyzing these methylated monosaccharides, the unmethylated hydroxyl group, which is the joint point of the original monosaccharide residue, can be obtained [31].

**Figure 6:**
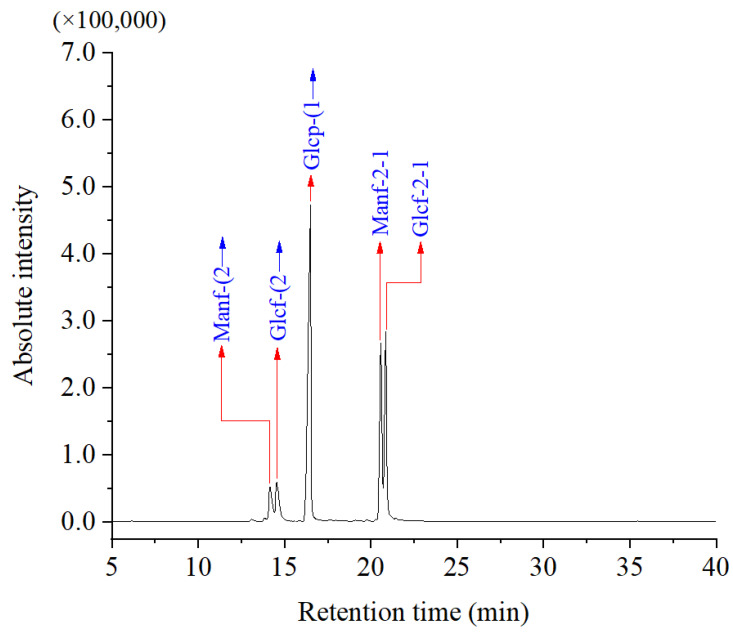
PMAAs results of methylation analysis of DPW.

Figure 6 and Table 2 show the linkage types and molar ratio percentages of DPW based on the Complex Carbohydrate Research Center (CCRC) spectral database (Complex Carbohydrate Structure Database, CCSD) for partially methylated alditol acetates (PMAA). DPW contained five main glycosidic linkages (as marked in red), namely 2-linked Manf, 2-linked Glcf, 1-linked Glcp, 1,2-linked Manf, and 1,2-linked Glcf, with molar percentage ratios of 0.031:0.038:0.585:0.17:0.177. Methylation experiments require sample reduction, and fructose can be easily converted into mannitol and glucitol [32]. Additionally, the monosaccharide analysis in Section 3.3 showed that only glucose and fructose were present in the DPW sample. Therefore, combined with the above data, it can be concluded that Manf glycosides were derived from the reduction of fructose.

### 3.6. ^1^H NMR, ^13^C NMR, and DEPT Analysis

**Figure 7:**
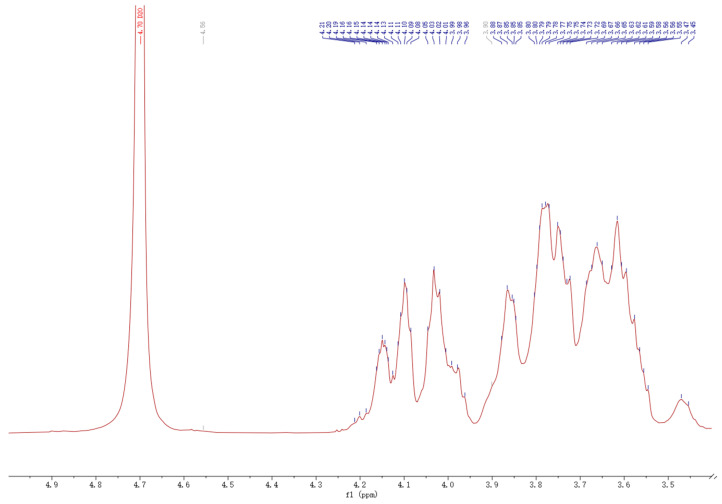
^1^H NMR spectrum of DPW.

The anomeric proton resonance region was first analyzed, as shown in Figure 7. The ^1^H-NMR spectrum of DPW revealed that it has a relatively weak signal peak at δ 5.32 ppm. Combined with the methylation analysis above, it can be seen that δ 5.32 ppm should be the anomeric hydrogen belonging to -α-D-Glcp-1→. However, there are strong signal peaks in the range of δ 3.5–4.2, and it is difficult to assign them according to ^1^H-NMR alone. Hence, other spectra are needed for accurate analysis.

**Figure 8:**
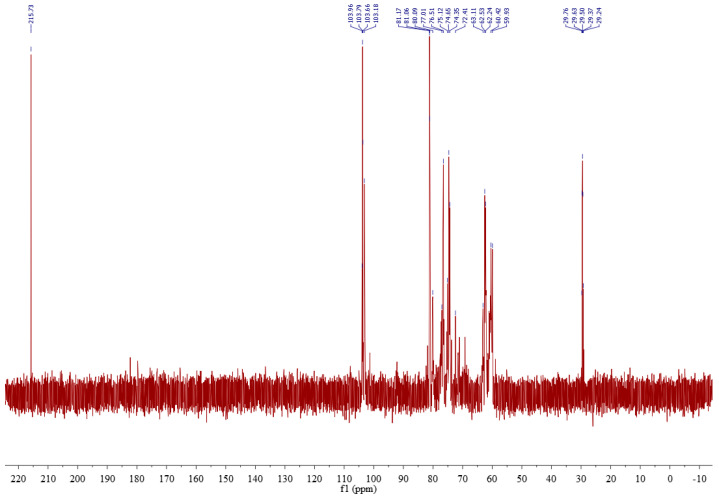
^13^C NMR spectrum of DPW.

The carbon spectrum research object is ^13^C, not the naturally abundant ^12^C, which requires more scans and time than the hydrogen spectrum. In the ^13^C NMR spectrum of DPW (Figure 8), there were two signal peaks, that is, δ 104.59 and δ 93.83 ppm, in the anomeric carbon signal region, and their peak area integration ratio was about 24:1. Therefore, δ 104.5 ppm can be said to belong to the main anomeric carbon signal of DPW. Combined with the methylation analysis results of DPW, δ 104.5 ppm was assigned to fructose residues, and δ 93.83 was assigned to glucose residues. Additionally, the anomeric signal of DPW showed a clear downward shift, indicating that DPW contains a β-glycosidic bond.

**Figure 9:**
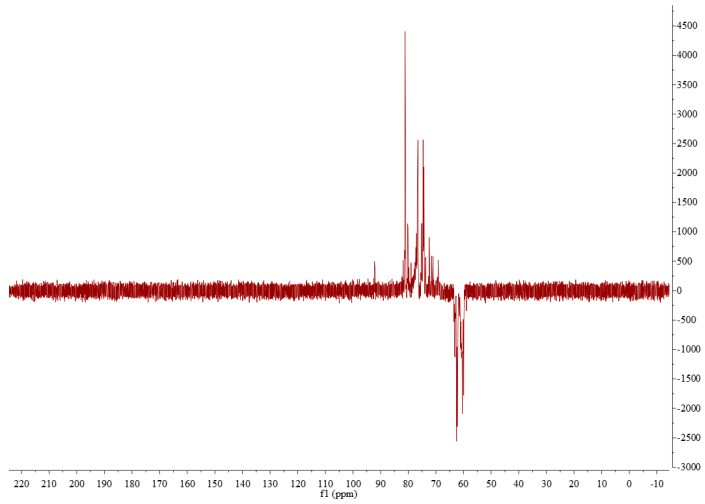
DEPT spectrum of DPW.

DEPT135 belongs to the carbon spectrum and is a very useful method for identifying primary, secondary, tertiary, and quaternary carbon atoms [33]. DEPT135 has different phases for the above four carbons: among them, the primary carbon signal -CH3- is upward; the secondary carbon -CH2- is downward; the tertiary carbon -CH- is upward; and the quaternary carbon -C- has no peaks. Thus, the DEPT135° spectrum can distinguish three carbon nuclei, of which the secondary carbon -CH2- is very easy to identify because it is the only one with a downward phase. By comparing the chemical displacement, primary carbon and tertiary carbon can also be distinguished. The DEPT135 of DPW is shown in Figure 9, and the methylene signal peak at δ 62.23 should be assigned to C1 of fructose residue and δ 63.47 to C6 of fructose residue.

### 3.7. ^1^H-^1^H COSY, HSQC, and HMBC Analysis

^1^H-^1^H COSY, HSQC, and HMBC belong to 2D NMR, and the spectral representation is different from that of 1D NMR, which only consists of points. The hydrogen spectrum and carbon spectrum only describe the information of a single atom, while the 2D NMR spectrum can link the information of the single atom, so as to determine the relative positional relationship of each atom. The ^1^H-^1^H COSY spectrum can connect hydrogen nuclei separated by three covalent bonds, such as -CHa-CHb-; the HSQC spectrum can connect hydrogen and carbon nuclei separated by one covalent bond, such as -CH-. Through these two methods, only the fragment information of the molecule can be obtained, and the information of the whole molecule can be obtained only by combining the fragment and fragment information. In this case, the HMBC spectrum can connect the hydrogen nucleus and carbon nucleus separated by two to three covalent bonds, such as -CHa-CHb-, and the peak position of Ha can correspond to the carbon of Hb. Here, the signal of hydrogen–carbon nucleus sites separated by two covalent bonds is usually stronger than that of hydrogen–carbon nucleus sites separated by three covalent bonds.

**Figure 10:**
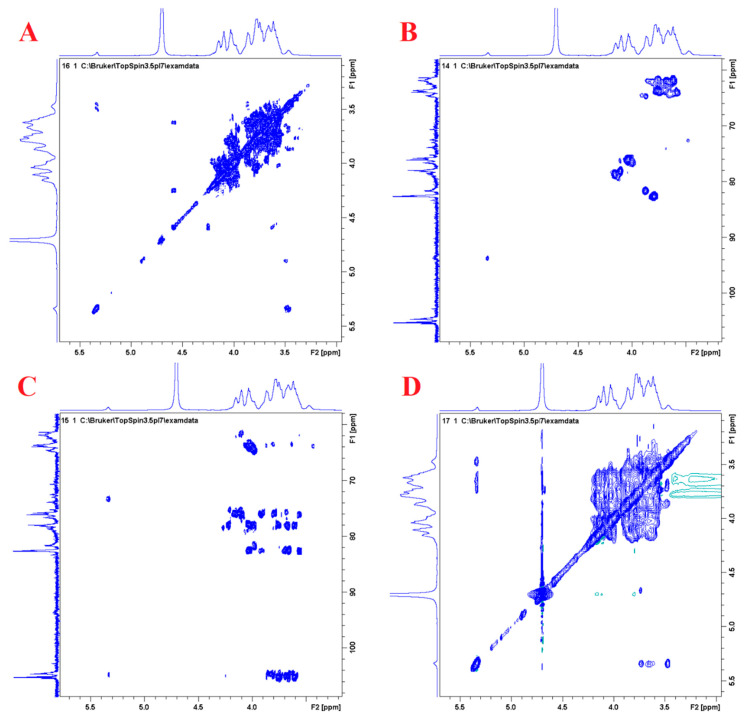
2D NMR spectra of DPW: (**A**) ^1^H-^1^H COSY; (**B**) HSQC; (**C**) HMBC; and (**D**) NOESY.

As shown in Figure 10A, in ^1^H-^1^H COSY, four groups of chemical shifts located at δ 5.32/3.44, δ 3.44/3.65, δ 3.65/3.36, and δ 3.36/3.75 denoted the correlations of H1–H2, H2–H3, H3–H4, and H4–H5 on glucose residues, respectively. Chemical shifts located at δ 4.15/3.99, δ 3.99/3.75, and δ 3.75/3.66 represented the correlations of H3–H4, H4–H5, and H5–H6 on fructose residues, respectively.

Through the analysis of the HMBC spectrum (Figure 10C) of the multi-bond carbon–hydrogen relationship of DPW, the arrangement sequence of fructose and glucose in its sugar chain was determined, and the assignment of ^1^H NMR and ^13^C NMR chemical shifts for the main residues of DPW in Table 3 was also checked for correctness. Studies have found that the typical feature of inulin-type fructans was the presence of glucose residues at the terminal positions [34]. For DPW, a strong cross-peak was located at H1 (α-D-Glcp-1)–C2 (β-D-Fruf-2,1), which indicated the existence of α-D-Glcp-1→2-β-D-Fruf-1→.

In addition, there was a cross-peak of H1a,1b (β-D-Fruf-2,1)–C2 (β-D-Fruf-2,1), which indicated that the fructose residues were linked by a 2,1-linkage mode. Meanwhile, H3 (β-D-Fruf-2,1)–C2 (β-D-Fruf-2,1) and H3 (β-D-Fruf-2,1)–C4 (β-D-Fruf-2,1) were also detected, as well as three cross-peaks of H4 (β-D-Fruf-2,1)–C6 (β-D-Fruf-2,1), H4 (β-D-Fruf-2,1)–C3 (β-D-Fruf-2,1), and H4 (β-D-Fruf-2,1)–C5 (β-D-Fruf-2,1), and those results were consistent with the methylation analysis results in Section 3.5.

In summary, it could be inferred that the chemical structure of DPW is α-D-Glcp-1→2-β-D-Fruf-1→(2-β-D-Fruf-1) _n_→.

### 3.8. PTIO Free Radical-Scavenging Activity

Studies have found that excessive reactive oxygen species (ROS) can cause damage to cells and induce various diseases [35,36]. Therefore, ROS scavenging plays a very important role in medicine, food, chemistry, and so on [37]. To date, many antioxidant evaluation methods and chemicals have been developed to quantify ROS-scavenging levels of different substances with antioxidant potential, among which DPPH free radicals and ABTS free radicals are most commonly used [38]. However, such radicals are nitrogen-centered radicals (reactive nitrogen species; RNS), not oxygen-centered radicals. For certain substances with antioxidant potential, it is scientifically unreasonable to evaluate ROS-scavenging levels by RNS-scavenging models [39]. Therefore, it is crucial to develop a reliable, stable, oxygen-centered free radical-scavenging method to test the level of ROS scavenging. For this purpose, 2-Phenyl-4,4,5,5-tetramethylimidazoline-1-oxyl 3-oxide free radical (PTIO·) is an ideal free radical centered on oxygen. PTIO· is a hydrophilic compound that is stable at room temperature. The elimination of PTIO free radicals can be easily detected directly with a common spectrophotometer and has been widely used in antioxidant activity evaluation.

**Figure 11:**
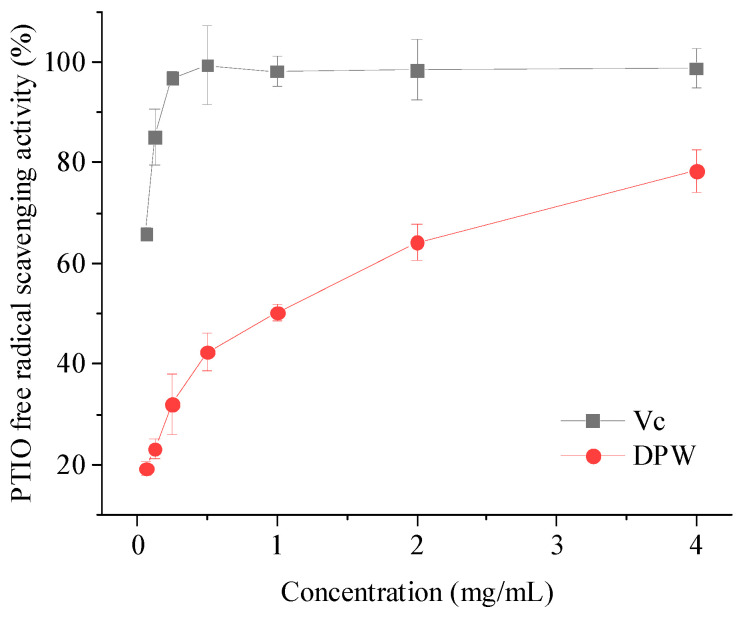
The scavenging activity of DPW and V_C_ on PTIO free radicals.

The scavenging efficiency regarding PTIO free radicals was positively correlated with DPW concentration, as shown in Figure 11. With the increase of DPW, the scavenging efficiency of DPW to PTIO· gradually increased and reached 81.24% at 4.0 mg/mL. Meanwhile, the IC_50_ of DPW to PTIO free radicals was 1.54 mg/mL. In our previous study, IC_50_ of DPW to DPPH free radicals and ABTS free radicals were all >4 mg/mL under the same dilution conditions [23], which indicated that DPW had better scavenging effects on ROS than RNS.

Researchers have found that the antioxidation of different polysaccharides is very closely related to their monosaccharide composition, glycosidic bond, molecular weight, chain conformation, and so on [40]. In general, the antioxidant activity of polysaccharides with relatively low molecular weight is more remarkable than that of relatively high molecular weight polysaccharides [41]. Furthermore, some scholars believe that the α-glycosidic bonds also affect antioxidant activity [42]. The DPW obtained in the present study belongs to the fructans group, and polysaccharides with similar structures have been widely demonstrated to have strong antioxidant effects [43,44]. However, the underlying relationships between the structure and the function of different polysaccharides are complex, and more studies are needed to interpret the dominant factors responsible for the antioxidant activity of DPW and other polysaccharides.

### 3.9. Adhesion Effect of Probiotic Strains under DPW Treatment

**Figure 12:**
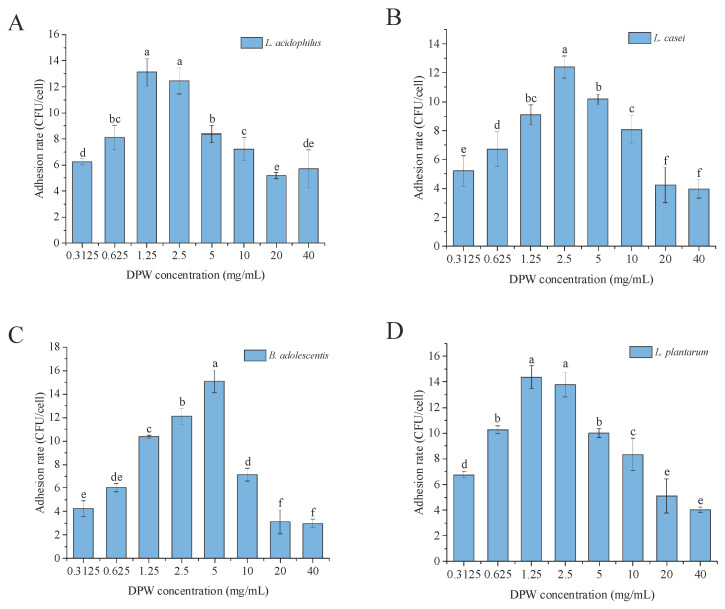
Effect of DPW treatment on probiotic strains adhesion ratio on Caco-2 surface. (**A**) for *L. acidophilus*, (**B**) for *L. casei*, (**C**) for *B. adolescentis*, and (**D**) for *L. plantarum.* Different letters on columns indicated a significant difference (*p* < 0.05).

The adhesion effects of probiotic strains treated with different concentrations of DPW are shown in Figure 12. As the concentration increased, the adhesion rate of four probiotic strains on Caco-2 cells showed a consistent trend of first increasing and then decreasing. The adhesion rate of all four probiotic strains reached its peak under DPW treatment of 1.25 to 5 mg/mL. DPW of lower or higher concentration was not conducive to the adhesion of probiotic strains on Caco-2 cells. The structure analysis showed that DPW isolated from daylily belonged to the fructans group. Studies have found that fructans are an excellent dietary supplement that is not digestible or fermentable and can enrich the abundance of lactic acid bacteria such as *Bifidobacterium* in the colon, thereby improving intestinal health [43]. Zhang et al. (2021) studied the intestinal health effect of *Polygonatum cyrtonema* and found that the fructan-type polysaccharides isolated from *P. cyrtonema* could promote the growth of *Bifidobacterium* and *Lactobacillus* strains (*p* < 0.05) [45]. Mueller et al. (2016) found that both β-2,1-linked inulin-type fructans and β-2,1- and β-2,6-linked branched mixed-type fructans could significantly enhance the growth of *Lactobacillus acidophilus* and *Lactobacillus paracasei* [46]. Additionally, Ramirez-Perez et al. (2023) found that the administration of linear (inulin) and branched fructans (agave fructans) could significantly increase *Lactobacillus* and *Bifidobacterium* abundance in the cecum of Wistar rats [47].

It has become a very common strategy to maintain the ecological balance of the intestine and avoid the occurrence of intestinal diseases by supplementing probiotics. In order to play a probiotic role, a certain level of probiotics must be maintained in the gastrointestinal tract. Therefore, compared with continuous supplementation, the colonization of the supplemented probiotics in the gut is crucial. Probiotics adhere to human digestive tract mucosal cells through some specific components on the surface, and adhesion is key to the colonization and proliferation of probiotics [48,49] in order to reject the adhesion and invasion of normal cells by pathogenic bacteria. In this study, DPW could promote the adhesion of the above four probiotics on the surface of Caco-2, which indicated that DPW could be used as a potential active ingredient to maintain intestinal health. However, its specific effect and mechanism of action need to be further studied, which is of great significance in developing functional products from daylily.

## 4. Conclusions

In this study, crude polysaccharides were first extracted from the edible *Hemerocallis* daylily (*Hemerocallis citrina* Baroni) flower by ultrasound-assisted extraction and alcohol precipitation. Combined with DEAE-Sepharose Fast Flow column chromatography, a novel water-soluble polysaccharide DPW was prepared for the first time. The molecular weights (*M*_p_, *M*_w_, and *M*_n_) of DPW obtained by HPSEC analysis were 2.275, 2.224, and 2.143 kDa, respectively. Monosaccharide composition analysis revealed that DPW comprised only two different monosaccharides, namely, glucose and fructose, in the molar ratio 0.242:0.75. Methylation analysis and NMR analysis revealed that DPW belonged to the fructans group, and its structure was α-D-Glcp-1→2-β-D-Fruf-1→(2-β-D-Fruf-1) _n_→. The antioxidant test results showed that DPW showed strong PTIO·-scavenging efficacy with IC_50_ of 1.54 mg/mL. Additionally, DPW of 1.25 to 5 mg/mL could significantly increase the adhesion rate of *L. acidophilu*, *L. casei*, *B. adolescentis*, and *L. plantarum* strains on Caco-2, indicating that DPW can be used as a potential active ingredient to maintain intestinal health.

The results of the present study provide a theoretical basis as well as practical support for the further development and comprehensive use of daylily polysaccharides as functional active substances. However, the unique and complex chemical structure of polysaccharides determines the complexity and difficulty of studying their chemical structure and structure–activity relationships. Although some progress has been made in these aspects in recent years, there are still many problems to be overcome in the study of the advanced structures and structure–activity relationships of polysaccharides. Therefore, it is still necessary for scientific and technological researchers to continue in-depth research in this area.

## Figures and Tables

**Table 1:** Molecular weights, polydispersity, and monosaccharide composition of DPW.

Parameters	Values
Molecular weight (kDa)	
Peak molecular weight (*M*_p_)	2.275
Number-average molecular weight (*M*_n_)	2.143
Weight-average molecular weight (*M*_w_)	2.224
Polydispersity index (PDI)	1.038
Monosaccharide composition (molar ratio)	
Glucose	0.242
Fructose	0.758

**Table 2:** Glycosidic linkage and peak area ratio of DPW.

Methylated Sugar	Mass Fragments (*m*/*z*)	Molar Ratios	Type of Linkage
1,3,4,5-Me4-Manf	87, 101, 129, 145, 161	0.031	Manf-(2→
1,3,4,5-Me4-Glcf	87, 101, 129, 145, 161	0.038	Glcf-(2→
2,3,4,6-Me4-Glcp	43, 71, 87, 101, 117, 129, 145, 161, 205	0.585	Glcp-(1→
3,4,6-Me3-Manf	43, 71, 87, 99, 101, 129, 145, 161, 189	0.17	→1)-Manf-(2→
3,4,6-Me3-Glcf	43, 71, 87, 99, 101, 129, 145, 161, 189	0.177	→1)-Glcf-(2→

Note: ‘→’ represented the possible linkage of methylated sugar residues.

**Table 3:** ^1^H NMR and ^13^C NMR chemical shift assignments of major residues.

Glycosyl Residues	H1a, 1b	H2	H3	H4	H5	H6a, 6b
	C1	C2	C3	C4	C5	C6
α-D-Glcp-1	5.32	3.44	3.65	3.36	3.75	3.71, 3.61
	93.76	72.59	73.98	70.59	75.74	61.67
(a) β-D-Fruf-2,1	3.80, 3.60	-	4.15	3.99	3.75	3.65, 3.73
	62.13	104.5	78.3	75.63	82.41	63.47
(b) β-D-Fruf-2,1	3.74, 3.60	-	4.08	3.85	3.84	3.91, 3.62
	61.28	105.2	78.18	73.01	81.68	64.53

## Data Availability

The original contributions presented in the study are included in the article and Supplementary Material, further inquiries can be directed to the corresponding author.

## Supplementary Materials

The following supporting information can be downloaded at: https://www.mdpi.com/article/10.3390/foods13050721/s1, Figure S1. Chromatography of eluted DPs on DEAE Sepharose Fast Flow Column.
