# Supplementary figures and images for: A Novel Water-Soluble Polysaccharide from Daylily (Hemerocallis citrina Baroni): Isolation, Structure Analysis, and Probiotics Adhesion Promotion Effect
Source: Foods. 2024 Feb 27;13(5):721. doi: 10.3390/foods13050721 (PMC10931500; doi:10.3390/foods13050721)

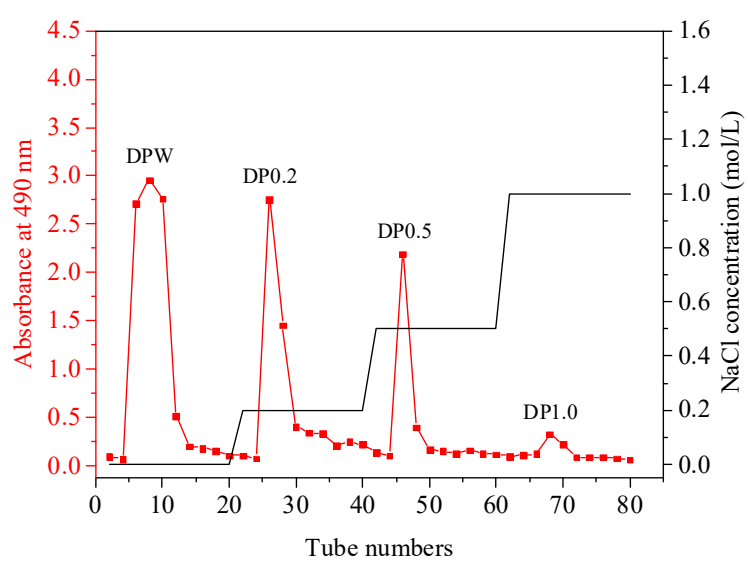

Figure S1 Chromatography of eluted DPs on DEAE Sepharose Fast Flow Column

Supplement: Supplementary file 1 [file foods-13-00721-s001.zip › foods-2858886-supplementary.pdf]
